# Mineralocorticoid Receptor, A Promising Target for Improving Management of Low Back Pain by Epidural Steroid Injections

**DOI:** 10.24015/JAPM.2016.0023

**Published:** 2016-07-26

**Authors:** Shaimaa I.A. Ibrahim, Judith A. Strong, Jun-Ming Zhang

**Affiliations:** 1 Pain Research Center, Department of Anesthesiology, University of Cincinnati College of Medicine, Cincinnati, USA; 2 Graduate Program in Molecular, Cellular, and Biochemical Pharmacology, University of Cincinnati, Cincinnati, USA

## Abstract

**Aim of review:**

Low back pain is a major health problem in United States and worldwide. In this review, we aim to show that mineralocorticoid receptor (MR) activation has a critical role in the initiation of immune and inflammatory responses, which in turn can impact the effectiveness of the currently used steroids for epidural injections in low back pain management since most steroids activate MR in addition to the primary target, glucocorticoid receptor (GR). Moreover, we would like to determine some of the benefits of blocking the MR-induced negative effects. Overall, we propose a novel therapeutic approach for low back pain management by using a combination of a MR antagonist and a GR agonist in the epidural injections.

**Method:**

We will first introduce the societal cost of low back pain and discuss how epidural steroid injections became a popular treatment for this condition. We will then describe several preclinical models used for the study of low back pain conditions and the findings with respect to the role of MR in the development of inflammatory low back pain.

**Recent findings:**

MR has pro-inflammatory effects in many tissues which can counteract the anti-inflammatory effects induced by GR activation. Blocking MR using the selective MR antagonist eplerenone can reduce pain and sensory neuron excitability in experimental models of low back pain. Moreover, combining the MR antagonist with clinically used steroids is more effective in reducing pain behaviors than using the steroids alone.

**Summary:**

MR antagonists are promising candidates to increase the effectiveness of currently used steroids. Since the activation of the MR is evident in preclinical models of low back pain, blocking its deleterious effects can be beneficial in managing inflammatory pain conditions.

Low back pain is a major health problem in United States and worldwide. In many cases, it can become chronic. Low back pain can be caused by many disorders such as lumbar herniated discs and compression of nerve roots. The clinical use of epidural steroid injections for low back pain management has increased dramatically during the past decade. However, this popularity doesn't reflect effectiveness. Clinical trials reported mixed results regarding the effectiveness as not all patients achieve adequate pain relief. Preclinical studies, on the other hand, largely focus on the effects of various clinically used steroids on the glucocorticoid receptor (GR), whereas the potential implications of mineralocorticoid receptor (MR) activation in low back pain and other inflammatory pain conditions have been largely ignored.

## Chronic Back Pain Cost to Society

Chronic pain continues to be an important worldwide heath problem. According to the International Association for the Study of Pain (IASP), pain is considered to be chronic if it lasts for at least 3 months (1). Generally, research studies use the standard of pain duration of 3 to 6 months to define chronic pain. The prevalence of chronic pain in US population is 30.7%, and is higher in females (34.3%) than males (26.7%). Importantly, the prevalence increases with age (2). Approximately 100 million adults in the United States are impacted by chronic pain. There are indirect costs as well as direct costs arising from chronic pain. For example, not only does pain require medical treatment, but it also decreases worker productivity, increasing the economic burden to society. It's reported that the total cost of chronic pain, including medical expenditures and reduced productivity, was approximately $560–$635 billion in 2010. Surprisingly, the annual costs of chronic pain has surpassed the annual costs of the diseases usually considered the most costly, such as heart disease, cancer and diabetes ($309 billion, $243 billion, and $188 billion, respectively) (3). Generally, the number of people affected by pain is greater than the number affected by cardiovascular disease, diabetes or cancer.

Low back pain is the major type of chronic pain reported, being more common than osteoarthritis, rheumatoid arthritis and migraine headache. Low back pain is one of the leading causes of disability in the United States and other countries. It progresses to being chronic in about 30% of cases. The direct medical costs in the United States are estimated to be $34 billion, with over $100 billion in lost wages and productivity per year. Therefore, it is a major contributor to health care costs (2, 4, 5).

## Epidural Steroid Injections for Low Back Pain

In 2002, interventional pain management was regarded as a specialty (6). Chronic pain management using interventional techniques has become very popular, including epidural steroid injections, facet joint interventions, disc procedures and nerve block. According to a Medicare analysis, there has been a dramatic increase in the use of interventional techniques. From 2000 to 2014, the increase was reported to be 153% in the Medicare population, with an annual rate of increase of 6.9% per 100,000 Medicare population (7).

Does the popularity of epidural steroid treatment reflect actual effectiveness or are they not really useful for management of chronic low back pain? In fact, the current medications may not be working appropriately. Not all of them succeed in relieving pain in chronic pain conditions like chronic low back pain (5). Given that inflammation is usually involved in low back pain, local injections of anti-inflammatory corticosteroids are commonly used to relieve both inflammation and pain. However, several randomized clinical trials showed that these medications failed to achieve adequate relief in many patients, which led to controversy regarding the effectiveness of steroid injections in pain management (8–12). For example, randomized clinical trials showed that epidural injections with local anesthetic alone improved chronic low back pain secondary to lumbar spinal stenosis in a comparable manner to the improvement due to epidural injections with steroids. These studies showed that there is no advantage for injecting steroids over local anesthetic (13). Other randomized clinical trials revealed that epidural steroid injections are only beneficial for a short time (8, 9).

## Preclinical Models for the Study of Low Back Pain

Low back pain can be detrimental as it reduces patients' quality of life. It can be caused by different disorders. For example, compression of nerve roots due to spinal stenosis (narrowing of spinal canal) or displacement or degeneration of the intervertebral disc is a major contributor to low back pain. Inflammatory conditions such as arthritis can be also involved. Treatment strategies range from surgeries (if possible and in severe cases) to the less invasive procedure of epidural steroid injections (4, 5, 14). The increased use of interventional techniques in pain management and the controversial results in clinical trials led to the need for further investigation to confirm the safety and effectiveness of these steroid injections. Several preclinical models of back pain have been developed to simulate different clinical conditions for low back pain. For example, an early model was developed in rats by applying nucleus pulposus (NP, the inner jelly-like material of the vertebral disc) into the lumbar dorsal root ganglia (DRG) (15). A different rat model is implemented by inducing local inflammation of the DRG (LID), which involves the local application of the immune stimulator zymosan in the vicinity of the L5 and/or L4 DRGs. These models can cause mechanical hypersensitivity, tactile allodynia and cold allodynia. In addition to the pain behaviors, these models cause upregulation of pro-inflammatory cytokines, satellite glial cell activation and increased sensory neuron excitability. As a result of the presence of materials not recognized as “self” elements by the immune system, such as the NP or the immune stimulator, robust inflammatory cell infiltration of the DRG occurs. This can last for a long time after the model is established (16, 17). Another rat model of chronic compression of the DRG (CCD) is implemented by compressing the lumbar DRG through inserting L-shaped metal rods into the intervertebral foramina. This rod remains in place for the duration of the experiment (18, 19). Despite the fact that an immune stimulus was not used in the CCD model, upregulation of pro-inflammatory cytokines, satellite glial activation, macrophage infiltration and behavioral sensitivity to anti-inflammatory drugs were observed. The cytokine profile was similar to that seen in the LID model (20–22). In addition, sensory neurons showed increased sensitivity to many pro-inflammatory cytokines after implementation of the CCD model (23, 24). It is documented that the NFκB pathway is involved in these models (25, 26) and one of the major mechanisms of steroid anti-inflammatory effects is suppression of this pathway.

## Non-Steroidal and Steroidal Anti-Inflammatory Drugs in Managing Low Back Pain

Given that inflammation is a key player in inducing low back pain, it is expected that anti-inflammatory drugs can relieve some of the pain symptoms. In the aforementioned models, inflammatory responses were observed such as activation of satellite glia cells, infiltration of macrophages, increase in pro-inflammatory cytokines and activation of inflammatory signaling pathways (15, 16, 27–30). In the models of ruptured disc, NP can compress the DRG or adjacent nerve roots and also act as an inflammatory stimulus. In addition, it acts as a source of pro-inflammatory cytokines. Interleukin-1β (IL-1β) and tumor necrosis factor α (TNF-α) have been found in the NP and are also thought to contribute to pain behaviors in different models such as the NP and the CCD models (28, 31–33).

Non-steroidal anti-inflammatory drugs can alleviate pain behaviors in different rodent back pain models when given systemically (17, 30) or administered by local or epidural injections (29, 34). However, steroidal anti-inflammatory drugs are more often used clinically, and are usually given locally into the epidural space to manage different low back pain conditions. The application can be done through different routes such as intraforaminal, caudal or interlaminar routes. Different time points and different routes can have significant effects on the results. For example, in a preclinical study using the rat CCD model, triamcinolone (a steroidal anti-inflammatory drug) decreased pain behaviors applied epidurally when given 3 days after the establishment of the model. However, it didn't reduce pain behaviors when given 10 days after the establishment of the model (35, 36).

## Steroid Receptors, Inflammation and Low Back Pain

**Figure 1:**
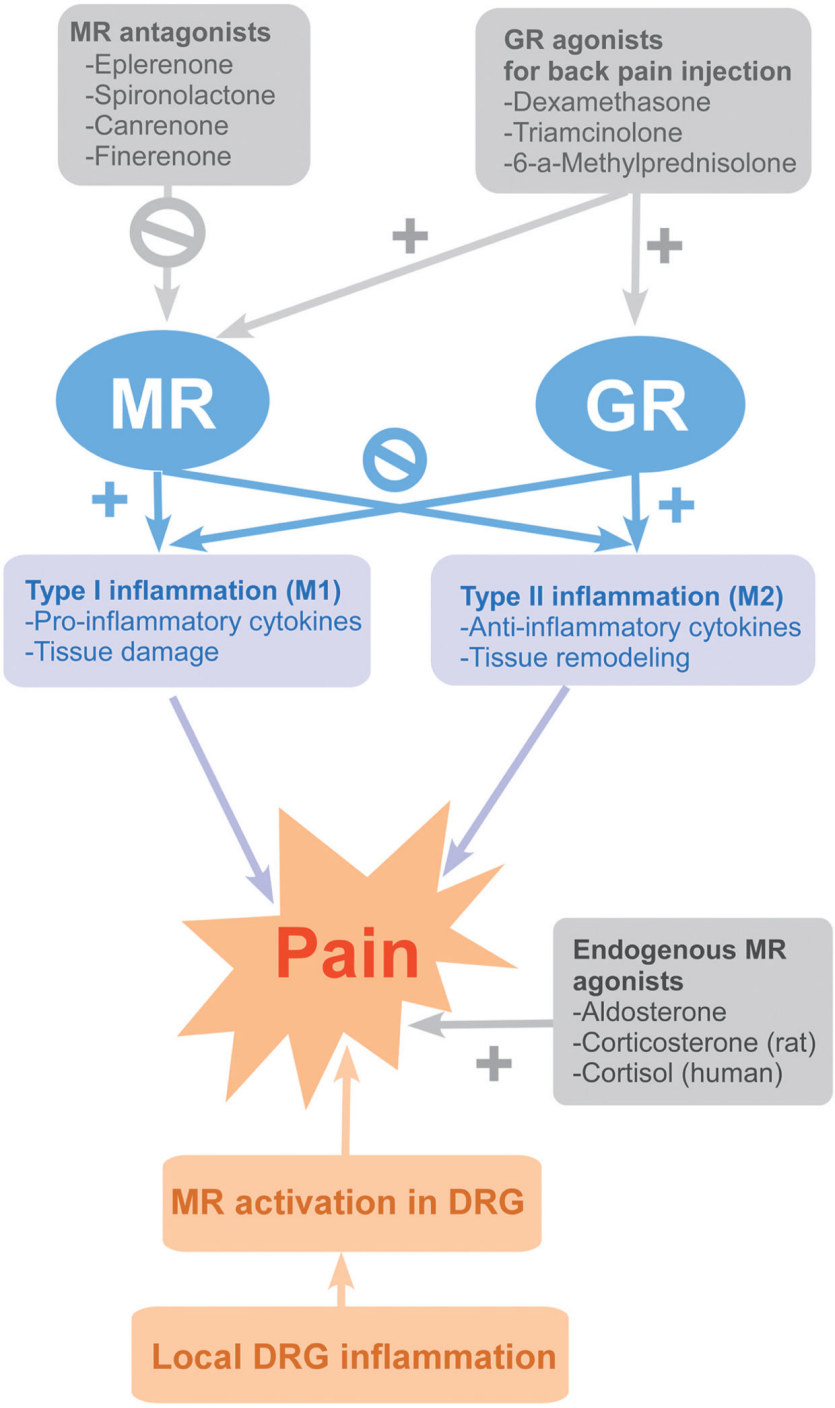
Diagram of Hypothesis About the Role of GR and MR in Mediating Effects of Clinically Used Steroids GR, glucocorticoid receptor; MR, mineralocorticoid receptor; DRG, dorsal root ganglia.

Clinically used steroids for back pain injections are meant to target the GR. GR is a member of the nuclear receptor family, for which the ligand diffuses into the cell and interacts with the receptor. Then, the ligand-receptor complex is translocated into the nucleus where it regulates gene expression. It is widely distributed in almost every tissue in the body. Its activation has an overall anti-inflammatory effect. As shown in Figure 1, GR activation stimulates type II inflammation (including M2 polarized macrophages) which involves tissue remodeling and wound repair. At the same time, it depresses type I inflammation (including M1 polarized macrophages) which involves tissue damage, high levels of oxidative metabolites and pro-inflammatory cytokines. Microarray analysis showed that 6 out of 10 selected M1 markers were upregulated after 3 days of LID (37).

Recently, it has been shown that some clinically used steroids (such as 6-α methylprednisolone and triamcinolone) can also activate the MR in vitro with significant potency (38, 39). The MR belongs also to the nuclear receptor family. Its function has been well-studied in the kidney, heart and hippocampal neurons. Additionally, in the central nervous system, MR is expressed in the glia (40). It was originally viewed only as the target of aldosterone. Its activation was thought to be mainly involved in electrolyte balance, specifically sodium and water reabsorption in kidney. However, this receptor was detected in other cell types including cardiomyocytes (41), brain neurons (42) and DRG neurons (43). In tissues other than kidney, MR activation has a pro-inflammatory role (promotes type I inflammation) that may offset the anti-inflammatory effects of GR activation (44, 45). MR activation is thought to contribute to inflammation in kidney, heart and central nervous system. In tissues other than kidney, glucocorticoids (the primary glucocorticoid in humans is cortisol, and in rodents is corticosterone), act as the primary endogenous activators of the MR. It is worth noting that in kidney, aldosterone is considered to be the only activator for MR due to the inactivation of glucocorticoids by 11β-dehydrogenase type 2 enzyme. In kidney, this enzyme inactivates the corticosterone (in rodents) and cortisol (in humans) in order to ensure that aldosterone will be the nominal activator of the MR to maintain the electrolyte balance. However, in non-renal tissues, due to reduced activity of the glucocorticoid-inactivating enzyme and the high corticosterone plasma concentration than aldosterone, corticosterone is considered to be the primary activator of MR (46).

## Preclinical Studies of Mineralocorticoid Receptor in Inflammatory Low Back Pain

Our focus in the laboratory is to explore the MR as a target for increasing the efficacy of clinically used steroids. We tested the hypothesis that MR is involved in the pro-inflammatory effects in the low back pain models, by combining a selective MR antagonist eplerenone with the current clinically used steroids, in order to block the MRmediated pro-inflammatory effects. In this way, MR activation and its antagonism of the desired anti-inflammatory effects caused by GR activation can be avoided.

**Figure 2:**
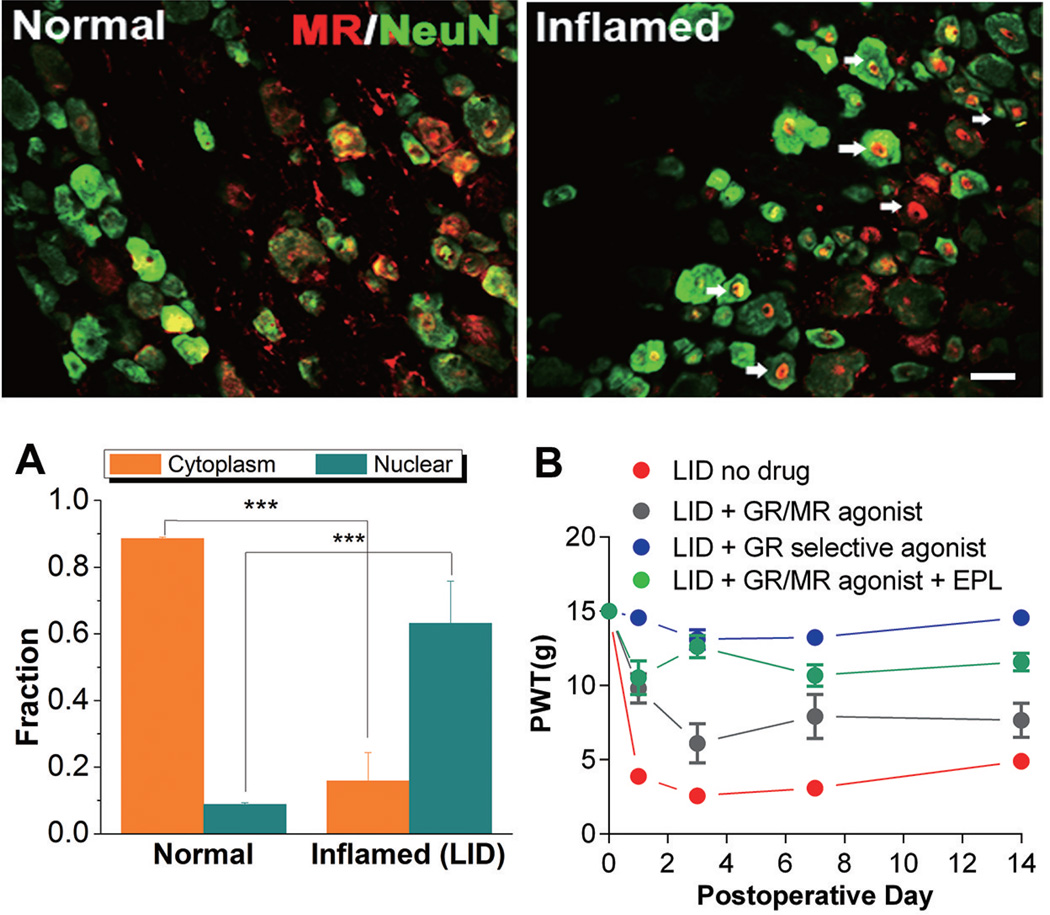
Changes in MR Expression and Effect of GR Agonists with and without MR Blocker, Eplerenone (EPL), on Pain Behaviors in Rats with Localized Inflammation of the DRG (LID) Two images above: Immunohistochemical staining showing nuclear translocation of activated MRs (red) in the inflamed DRG neurons (green) on POD 1. Scale bar=50 µm; A: Comparisons of the fraction of MRs in cytoplasm and nuclear between normal and LID neurons. ^***^p<0.001, student’s t-test. B: Effect of locally applied fluticasone (blue), 6-methylprednisolone alone (grey) or combined with eplerenone (“EPL”, green). Paw withdrawal threshold (PWT) was detected using von Frey testing; baseline plotted on POD 0. On each day, all points with nonoverlapping SEM differ significantly. N=6 or more rats/group. GR, glucocorticoid receptor; MR, mineralocorticoid receptor; DRG, dorsal root ganglia; LID, localized inflammation of the DRG; EPL, eplerenone.

We first examined MR expression in the inflamed and normal sensory ganglia. As shown in Figure 2, MR is present mainly in the cytoplasm in normal DRG neurons and rapidly translocated to the nucleus after DRG inflammation. Nuclear translocation, which reflects MR activation, is observed after 1 day of L5 DRG inflammation. The peak of nuclear localization was observed at 1 day after the LID with gradual reduction until returning to the normal distribution after 14 days. To confirm that MR nuclear localization was not due to systemic effects of the model, DRGs were taken from the T12 level some distance from the inflamed L5. This remote DRG did not show any changes in MR nuclear localization. In addition, a different MR antibody was used (directed against A/B region in the N-terminal) to confirm the MR cytoplasmic localization in normal DRG. Thus, this MR nuclear localization was specific to the local inflammation of the L5 DRG. Moreover, immunohistochemistry experiments using glial fibrillary acidic protein (GFAP) marker (marker of glia activation) showed that local eplerenone application to L5 DRG was able to reduce satellite glia activation in the inflamed DRG, which may contribute to the anti-inflammatory effects of eplerenone (43).

In behavioral studies using the LID model, where the L5 DRG is inflamed with the immune activator zymosan in incomplete Freund's adjuvant (IFA), mechanical hypersensitivity was examined. Combining the MR antagonist (eplerenone) with the zymosan/IFA, allowing its local application starting at the time of L5 DRG inflammation, reduced mechanical hypersensitivity as well as the pain duration (43). Moreover, eplerenone given orally starting 7 days after LID was able to reduce the mechanical hypersensitivity and cold allodynia. With the same oral route, eplerenone was effective in reducing pain behaviors when the administration started at the time of DRG inflammation (47). Oral eplerenone taken at the same time of LID was also able to reduce spontaneous activity of sensory neurons to normal levels. In addition, it reduced the sensory neuron hyperexcitability in locally inflamed DRG (47).

In an attempt to test the hypothesis that MR receptor activation played a role in the effectiveness of steroid drugs, the GR selective agonist (fluticasone) or GR/MR agonist (6-α methyl prednisolone) was locally applied to the inflamed DRG. In vitro studies showed that fluticasone activated only the GR, not the MR or other nuclear receptors (48), while 6-α methyl prednisolone, one of the popular steroids used for back pain injections, activated GR and MR with similar potency (38). Local fluticasone application ameliorated the pain behaviors induced by the LID model (Figure 2). Furthermore, it also normalized the spontaneous activity and reduced the hyperexcitability of sensory neurons induced by the LID model. On the other hand, 6-α methyl prednisolone led to a more modest improvement to the pain behaviors in the LID model, and the effect was not long-lasting. The sensory neuron hyperexcitability but was less reduced than with fluticasone, and there was a trend towards reducing spontaneous activity that did not reach significance.

However, when 6-α methyl prednisolone was applied to the DRG in combination with eplerenone to block the MR effects, the pain behaviors were greatly improved (Figure 2) (47). Therefore, we can infer that the effects of GR selective agonist fluticasone were stronger and more long-lasting than that of 6-α methyl prednisolone which activates both GR and MR. Most importantly, by eliminating MR activation, we were able to improve efficacy of less selective GR agonists.

### Pro-nociceptive Effects of the Mineralocorticoid Receptor

MR has pro-nociceptive effects in the DRG neurons directly through the activation of nuclear factor kappa B (NF-κB) transcription factor, which is known to be involved in pro-inflammatory effects in the DRG (49, 50) and other tissues (51–53). Application of different doses of aldosterone (the natural agonist of MR) to cultured DRG neurons isolated from normal animals led to dose-dependent effects on the number of action potentials evoked by current injection (43). Effects of MR activation on sensory excitability were further confirmed by the finding that increased excitability of small-diameter sensory neurons at 1 day after LID could be partially reversed by in vitro eplerenone application (after 8–12 hours in acute primary culture) (43). Another possible contributor to the pro-nociceptive effects was that MR activation in macrophages promoted a pro-inflammatory phenotype (46).

## Clinical Implications

It can be inferred from our findings that MR is expressed in the normal and inflamed DRG and it is translocated to the nucleus 1 day after LID. Furthermore, MR activation can mediate increased excitability in DRG neurons. Many other studies demonstrated that MR has pro-inflammatory effects in several tissues. Thus, the MR can be a promising target for low back pain management.

The choice of eplerenone in many of our preclinical studies is because of its high selectivity for MR over GR. The fact that it is a more selective MR antagonist than the previous generations of antagonist such as spironolactone, making it more appealing to be used as targeted therapy with high selectivity. In addition, it has been approved by FDA and is currently being used for treatment of hypertension and heart failure (54). Therefore, based on our findings, we propose a new therapy for managing inflammatory pain conditions including low back pain by combining eplerenone with the epidural steroid injections to improve their effectiveness.

Previous studies (48) showed that fluticasone propionate is more specific for the GR than most other steroids. Using the highly selective GR agonist fluticasone for epidural steroid injections would be an ideal approach for managing low back pain. However, fluticasone is mainly used in the form of nasal spray and aerosol for treatment of allergies and asthma. It has not been approved for use as an epidural injection. Among betamethasone, 6-α-methylprednisolone and triamcinolone currently most commonly used for epidural steroid injections, it is recommended that betamethasone may be most effective in managing low back pain based on previous studies (38) by comparing MR and GR agonist properties of different steroids used in clinical practice.

In other pain conditions such as arthritis (kneestoes) and painful joint inflammation, steroid injections are still used as a popular treatment to alleviate pain and inflammation. Based on recent findings from our study, MR blockers alone or in combination with GR agonists may also be beneficial for these conditions as MR is widely expressed in neuronal and non-neuronal tissues including the immune cells such as macrophage.

## Summary

Most low back pain conditions involve inflammation. Epidural steroid injections are very common treatment options. However, they fail to work in many patients. This can be due to the fact that most clinically used steroids can activate both GR and MR with significant potency. Since MR is involved in the inflammatory response in low back pain, using the current medications would not achieve the maximum efficacy because both receptors are activated. Hence, blocking the undesirable effects of MR activation by combining a MR antagonist with the clinically used epidural steroids may achieve higher efficacy than using the current steroids alone. This can open a new window for more targeted therapy and more relief for low back pain, which if successful will have a huge economic and social impact on society.
